# Advanced retinal disease detection using RHT-Net: a hybrid deep learning approach with augmented fundus imaging

**DOI:** 10.3389/fphys.2026.1837336

**Published:** 2026-06-24

**Authors:** Yaoyang Cheng, Yiling Pan, Guodao Zhang, Wei Wu, Yisu Ge, Jing Wang

**Affiliations:** 1 Institute of Intelligent Media Computing, Hangzhou Dianzi University, Hangzhou, China; 2 Fujian Key Laboratory of Big Data Application and Intellectualization for Tea Industry, Wuyi University, Wuyishan, China; 3 Department of Ophthalmology, Zhongshan Hospital, Fudan University, Shanghai, China

**Keywords:** convolutional neural networks, hybrid deep learning, retinal disease classification, RHT-Net, transformer architectures

## Abstract

**Introduction:**

Retinal diseases are a leading cause of visual impairment worldwide, highlighting the need for accurate and scalable automated screening systems. This study introduces RHT-Net (Retinal Hybrid Transformer Network), a novel hybrid deep learning architecture designed for multi-class classification of nine retinal diseases from color fundus images by combining the strengths of convolutional neural networks and transformer encoders.

**Methods:**

RHT-Net integrates residual convolutional neural networks for local feature extraction with transformer encoders to capture long-range global dependencies. A real-world dataset comprising 5,318 color fundus images collected from Bengali patients was used in this study. Data augmentation techniques, including rotation, flipping, and Gaussian noise addition, expanded the dataset to 21,272 images. Images were preprocessed through resizing to 224 × 224 pixels and contrast enhancement using Contrast Limited Adaptive Histogram Equalization (CLAHE). The augmented dataset was divided into training (80%) and testing (20%) subsets for model development and evaluation.

**Results:**

The proposed RHT-Net achieved a training accuracy of 97.93% with an F1-score of 96.10%. On the testing set, the model attained an accuracy of 96.12% and an F1-score of 92.28%. The overall classification performance reached an accuracy of 97.57% and an F1-score of 95.31%. Comprehensive evaluations, including class-wise performance analysis, confusion matrices, and Grad-CAM visualizations, demonstrated the model’s strong predictive capability, robustness, and interpretability across the nine retinal disease classes.

**Discussion:**

The findings indicate that RHT-Net is a promising and scalable approach for early retinal disease screening. By effectively capturing both local and global image features, the model achieves high classification performance while providing interpretable predictions. These characteristics support its potential integration into telemedicine and remote diagnostic workflows. However, further external validation and deployment-oriented optimization are necessary before real-world clinical implementation.

## 1 Introduction

Retinal diseases—diabetic retinopathy, glaucoma, macular scarring, optic disc oedema, central serous chorioretinopathy (CSCR), retinal detachment, retinitis pigmentosa, and myopia—are leading causes of preventable blindness worldwide. In impoverished parts of the world like Bangladesh, blindness is especially disturbing, with 1.5% of adults suffering from blindness and 21.6% suffering from impaired vision. The few ophthalmologists and the lack of access to devices contribute to delays to early diagnosis, early treatment, and irreversibility of blindness. This is a significant issue because early diagnosis and treatment with the use of AI in medicine can alleviate global visual impairment burden and allow for a better quality of life and economic contribution of a person.

There are clear benefits of artificial intelligence (AI) in medicine, especially for ophthalmologists. AI can help automate the reading of medical images, thus expediting the diagnosis process and improve accuracy within the process while relieving the time burden placed on busy clinicians. In general practice AI can help with predictive modelling, individual treatment plans, and resource distribution in areas of need. In the context of detecting retinal disease, AI systems have been shown capable of performing at the same or higher levels than experienced ophthalmologists at tasks such as screening for diabetic retinopathy and age-related macular degeneration. All of these offer advantages that improve diagnostic accuracy through deep learning algorithms, predictions about risk earlier as AI recognizes patterns in data, and improved follow-up with patients with telemedicine solutions. In addition, AI can help with some degree of socioeconomic disparity or over-treatment of telemedicine by offering low-cost screening at remote sites, improving treatments, and facilitating the integration of other at-home monitoring technology for ongoing patient management. The research presented in this paper has useful applications in clinical settings, such as automated screening programs for high-risk populations, telemedicine-based healthcare systems for rural patients, and decision-support systems for ophthalmologists and similar providers. By providing reliable multi-class classification, the critical task of early intervention and prevention of vision loss is achievable and substantially reduces the cost of providing vision healthcare in constrained resource settings. The main contributions of this study are summarized as follows:

1. We propose RHT-Net (Retinal Hybrid Transformer Network), a novel and carefully balanced hybrid architecture that synergistically integrates four residual CNN blocks for fine-grained local lesion feature extraction with exactly three transformer encoder layers for modeling long-range global dependencies in retinal images. This design effectively addresses the limitations of pure CNN models in capturing global context and pure Transformer models in terms of computational efficiency and data requirements, while maintaining approximately 25.5 million trainable parameters suitable for clinical deployment.
2. We introduce and utilize a unique Bengali fundus image dataset consisting of 5,318 original high-resolution color fundus images collected from two hospitals in Bangladesh. After stratified splitting and targeted augmentation, the dataset is expanded to 21,272 images covering nine clinically important retinal conditions from an underrepresented population.
3. We achieve state-of-the-art performance with 96.12% test accuracy and 92.28% F1-score on the held-out test set, while providing clinical interpretability through Grad-CAM visualizations. The work also emphasizes practical applicability in low-resource settings and telemedicine scenarios.

## 2 Related work

Deep learning applications for detecting, classifying, and grading retinal disease have grown quickly over the last several years. Initial approaches generally relied on standard convolutional neural networks (CNN) using local pattern-based models that were successful in retaining details such as lesions, vessels, and exudates. However, CNNs have limited capabilities in modeling long-range dependencies (and in some instances, with multi-label cases of patients with multiple co-existing retinal diseases), which led to the development of CNN and transformer hybrid architectures and attention-based models to address issues across local and global representations.

Sivaz and Aykut developed a multi-label retinal disease multi-label classifier based on Swin Transformer V2 with the unique Shunted Cross-Attention (SCA) head convolutional window, with the goal of better capturing supervised label correlations for the classification objective. They also applied Sharpness-Aware Minimization (ASAM) as well as a new loss function (SNDL) and improved the performance of a multi-label output strategy with the ODIR dataset (Sivaz and Aykut, 2025). Then Shen et al. developed a Structure-Oriented Transformer (SoT) for the OCT-based grading of retinal diseases. They had both a structure orientation module that emphasized retinal layers and a token voting system that combined feature representations from multiple patches (Shen et al., 2023). Ashoka developed EffiViT, which applied EfficientNet for local feature extraction with a Vision Transformer for long-range dependencies and outcome a superior result on the MuReD dataset, despite class imbalance challenges (Ashoka, 2025). Expanding on this trend, Lai and Liu proposed a CNN-ViT hybrid that combined Inception-ResNet-v2 features with a transformer encoder. They improved generalization using their model compared to either CNNs or ViT alone (Lian and Liu, 2024). Also Bi et al. developed MIL-ViT combining MIL with a vision transformer to learn patterns better in small lesions, proving especially successful in identifying early diabetic retinopathy (DR) and age-related macular degeneration (AMD) with several benchmark datasets (Bi et al., 2023). Zhou et al. constructed a ResNet50 model to address class imbalance in the data, applying multi-dimensional attention and an adaptive scale discriminator. They also introduced a combined loss hybridizing BCE and focal loss as the final loss classification (Zhou et al., 2024).

Other studies have explored ensemble learning, and explicability. For example, Singh et al. proposed a CNN – Transformer ensemble model that dynamically selects informed patches as well as integrating clinical domain knowledge, to produce successes in multi-disease classification (Singh et al., 2025). Liu et al. proposed STMF-DRNet, which is a multi-branch Swin Transformer architecture specifically for diabetic retinopathy grading. Rather than treating the whole image as an input to the classification model, they utilized object localization and patch-level attention to improve stage classification accuracy in real-world scenarios (Liu et al., 2025). Faria et al. showcased the significance of transparency by exploring a series of CNN backbones for classification and segmentation networks used for analyzing retinal vessels, further employing a combination of understandable AI (XAI) techniques like Grad-CAM and LayerCAM within their application framework (Faria et al., 2024).

Multiple publications took steps towards improving interpretability. Xu et al. combined EfficientNet and Swin Transformer to classify DR subtypes with high accuracy and with strong explanations using class activation maps (Xu et al., 2024). Djoumessi and Berens created a self-explainable hybrid CNN–Transformer that employed dual-resolution self-attention and class evidence maps to provide interpretable predictions focused on lesions while maintaining state-of-the-art levels of performance (Djoumessi et al., 2025). Gu et al. also presented a Swin transformer, SwinECAT, which embedded efficient channel attention in a Swin transformer backbone, which obtained strong results on nine-class retinal disease classification (Gu et al., 2025). Beyond hybrid models, ensemble and temporal models have also been created. AlMohimeed created MST-EDS, a multi-stage framework that incorporated ViT, DeiT, and Swin Transformer with PCA-based feature reduction and ensemble stacking. The use of ensemble stacking raised the classification accuracy of four major eye diseases significantly (AlMohimeed, 2025). In the temporal modeling space, Sushith et al. proposed a CNN–RNN hybrid model with attention that aimed to capture the disease progression in DR. Their model used sequential scans, which supported early detection of the disease. In their model, they used 9 images from 3 sequential scans, which achieved better sensitivity and specificity than traditional models (Sushith et al., 2025). Finally, Wang et al. presented a vision transformer model that is purely a vision transformer model for multi-label classification of retinal diseases. Their model architecture avoided convolutional modules entirely and instead solely relied on self-attention to model global and local dependencies. Within the ODIR-2019 dataset, their model outperformed state-of-the-art CNN approaches in multi-label accuracy and F1 score (Wang et al., 2024).

All together, these studies provide directions for several relevant areas of research. First, hybrid CNN–Transformer designs have been shown to outperform both CNNs and transformers alone by combining their underlying strengths in capturing both fine-grained vision information and contextual global context (Bi et al., 2023; Shen et al., 2023; Zhou et al., 2024; Lian and Liu, 2024; Xu et al., 2024; Gu et al., 2025; Djoumessi et al., 2025; Ashoka, 2025; Sivaz and Aykut, 2025). Second, ensemble methods and stacking approaches tend to improve robustness and generalizability across different datasets (AlMohimeed, 2025; Singh et al., 2025). Third, interpretability is becoming an increasingly important dimension, and whole solution frameworks are being presented with inherently explainable mechanisms rather than just on *post-hoc* (Faria et al., 2024; Djoumessi et al., 2025). Fourth, while temporal modeling of disease progression has not been extensively explored, it has great promise in diseases such as DR (Sushith et al., 2025). Lastly, multi-label classification is an important research direction mirroring the world risk of co-occurring diseases (Wang et al., 2024; Sivaz and Aykut, 2025). Recent studies have continued to advance hybrid and privacy-aware approaches in medical imaging. Sathiya and Tamilvizhi (2025) (Sathiya and Tamilvizhi, 2025) proposed a hybrid CNN-ViT model for efficient retinal disease screening on EyePACS and ODIR-5K datasets, achieving 97.9% accuracy through effective preprocessing and random oversampling to address class imbalance. Jayalakshmi and Tamilvizhi (2025) (Jayalakshmi and Tamilvizhi, 2025) introduced FedDRNet, a federated learning framework based on an efficient cross-stage recurrent model for privacy-preserving diabetic retinopathy prediction, attaining over 98% across multiple metrics while ensuring data security. Additionally, Sherine et al. (2025) (Sherine et al., 2025) developed Graph-Enhanced Transformers to model complex drug-drug interactions, demonstrating the capability of transformer-based architectures in capturing relational dependencies in biomedical tasks. Recently, Khan et al. (2025) (Khan et al., 2025) proposed a novel deep learning framework consisting of a Densely Connected Multidilated Convolution Neural Network (DCM-CNN) for capturing global contextual information and a Local-Patch-based CNN (LP-CNN) for extracting fine-grained local features. These two modules are combined through a synergic network to improve retinal disease classification on the RFMiD and ODIR-5K datasets. While their dual-branch approach effectively addresses contextual and local feature extraction, it relies entirely on convolutional operations and does not incorporate transformer-based global attention mechanisms.

Although recent hybrid CNN-Transformer models have demonstrated promising results in retinal disease classification, several important gaps remain. Most existing studies focus primarily on binary or limited multi-class classification tasks, utilize well-curated public datasets from Western populations, and pay limited attention to deployment challenges in low-resource clinical environments. Furthermore, issues such as severe class imbalance, real-world image quality degradation, and the need for clinical interpretability are often insufficiently addressed. In contrast, the proposed RHT-Net introduces a balanced residual CNN-Transformer architecture specifically optimized for nine-class retinal disease classification. It incorporates targeted data augmentation strategies and CLAHE preprocessing tailored for real-world fundus images, utilizes an underrepresented Bengali patient cohort, and integrates Grad-CAM-based interpretability. This unique combination of architectural balance, dataset diversity, practical preprocessing, and clinical orientation distinguishes the present work from existing literature and contributes toward the development of scalable, equitable, and deployable artificial intelligence solutions for retinal disease screening in developing countries.

## 3 Methodology

### 3.1 Dataset

In this study, we utilized a dataset consisting of 5,318 original high-resolution color fundus images. Nature offered these outstanding images organized to emphasize nine different classes of retinal pathologies, excluding Pterygium, to focus on pathologies affecting the eye’s posterior segment. Images were collected between July 2023 and February 2024, as a collaboration with the Health Informatics Lab at Daffodil International University and the representatives of two hospitals in Faridpur Bangladesh, the Anawara Hamida Eye Hospital, and B.N.S.B. Zahurul Haque Eye Hospital (Sharmin et al., 2024). The dataset is significant as it is the only dataset to our knowledge, representing the Bengali ethnic community. Images were taken on the TL-211 and TRC-50DX Topcon fundus cameras that allow high-resolution imaging, enabling fine details of the retinal structure. The original images received, had their resolution ranging from 3900×2600, to 2004 ×1690 but were standardized to JPG format at 2004 x 1690 pixel resolution for compatibility and consistency across deep learning frameworks. This preprocessing step along with a large sample size and diverse pathologic representation makes the dataset a useful resource for developing and validating automated diagnostic systems in ophthalmology.

The dataset is divided in the following nine classes and has the following number of images per class.

1. Central Serous Chorioretinopathy (CSCR) (101 images): A condition wherein fluid leaks and accumulates underneath the retina, causing a blister-like bulge in the retina, and typically blurry or distorted central vision. It occurs most often in young to middle-aged adults.
2. Diabetic Retinopathy (1,509 images): A complication of diabetes that involves the retina’s blood vessels leaking, oozing and swelling (macular oedema), or growing other blood vessels in an abnormal fashion (proliferative retinopathy). Vision loss can result if left untreated.
3. Optic Disc Edema (127 images): The swelling of the optic disc may be due to intracranial pressure, inflammation, or vascular issues. There may be a headache and/or blurred vision. This could represent a more serious systemic disorder.
4. Glaucoma (1,349 images): A series of disorders involving impairment of the optic nerve that is often associated with rising intraocular pressure. Glaucoma can result in the progressive loss of peripheral vision, with advanced disease leading to tunnel vision and even total blindness.
5. Healthy (1,024 images): Normal retinal images with no evidence of disease. These images are a control to distinguish healthy retinal images from those that are diseased.
6. Macular Scar (444 images): Fibrotic scarring occurs at the macula; the middle of the retina and responsible for detailed vision. The most common causes of this scarring are age-related macular degeneration (AMD) or trauma. Once a macular scar has formed, the result is permanent central vision loss.
7. Myopia (500 images): Pathological high myopia (degenerative myopia) occurs when the eyeball elongates (causing thinning), when there is some sort of retinal tear or detachment, which might cause blurred distance vision and puts the patient at increased risk of complications.
8. Retinal Detachment (125 images): Retinal detachment occurs when the retina pulls away from the supportive tissues. Symptoms of a retinal detachment can include sudden vision loss sometimes accompanied by bright flashes or floaters. This needs to be treated right away to avoid permanent blindness.
9. Retinitis Pigmentosa (139 images): An inherited disorder that causes the retinal photoreceptor cells to slowly break down, leading to night blindness, tunnel vision, and eventually severe vision loss.

**Figure 1:**
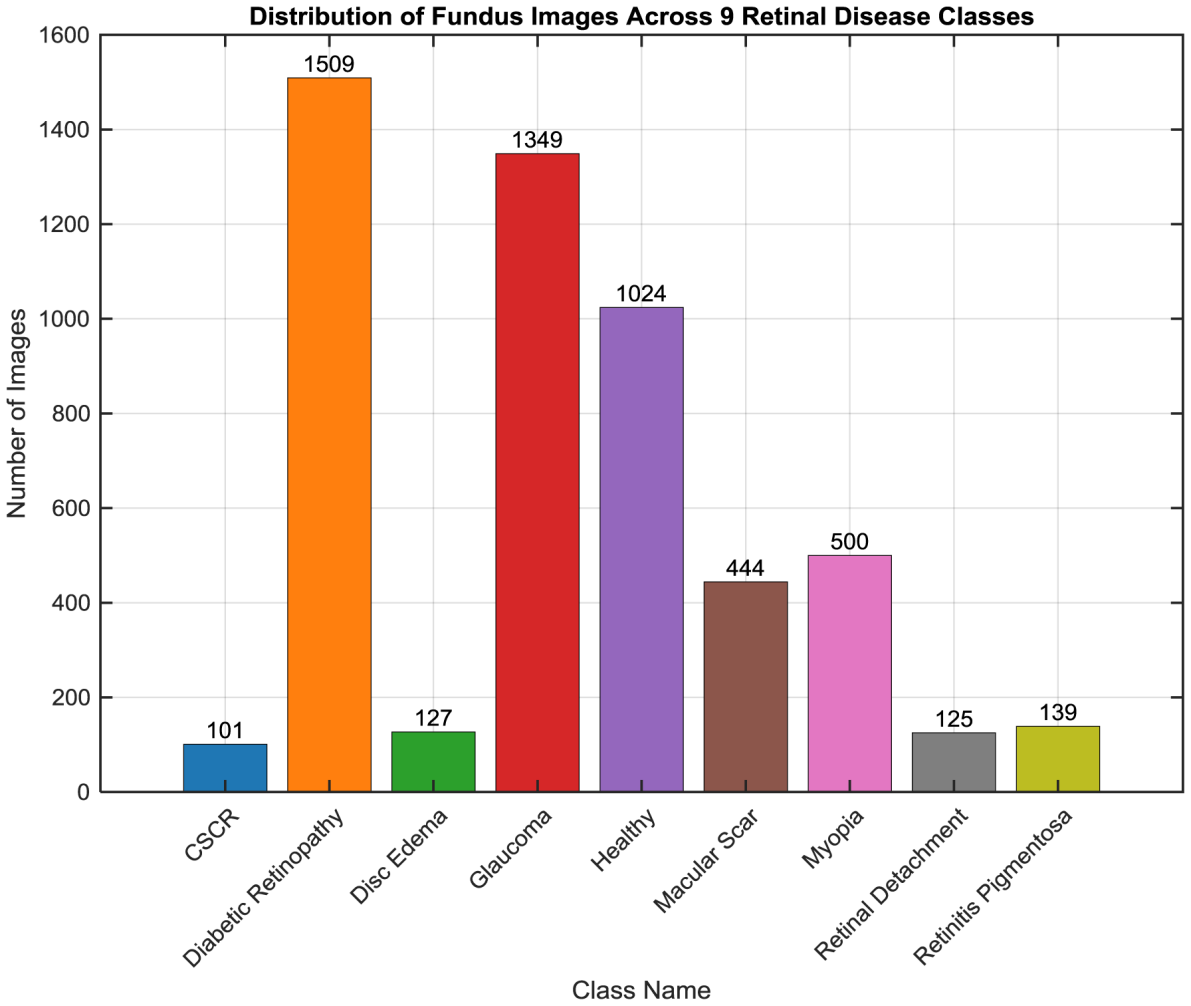
Distribution of fundus images across 9 retinal disease classes.

This distribution of the conditions demonstrates class imbalance, with some classes like Diabetic Retinopathy and Glaucoma having a much larger presence than rarer conditions like CSCR and Retinal Detachment. **Figure 1** clearly represents this distribution through a bar chart, with each class represented by a bar that is color-coded, as well as axis designations where the y-axis indicates the number of images, and the x-axis lists the class names. The overview highlights the importance of data augmentation to achieve more balance in training.

**Figure 2:**
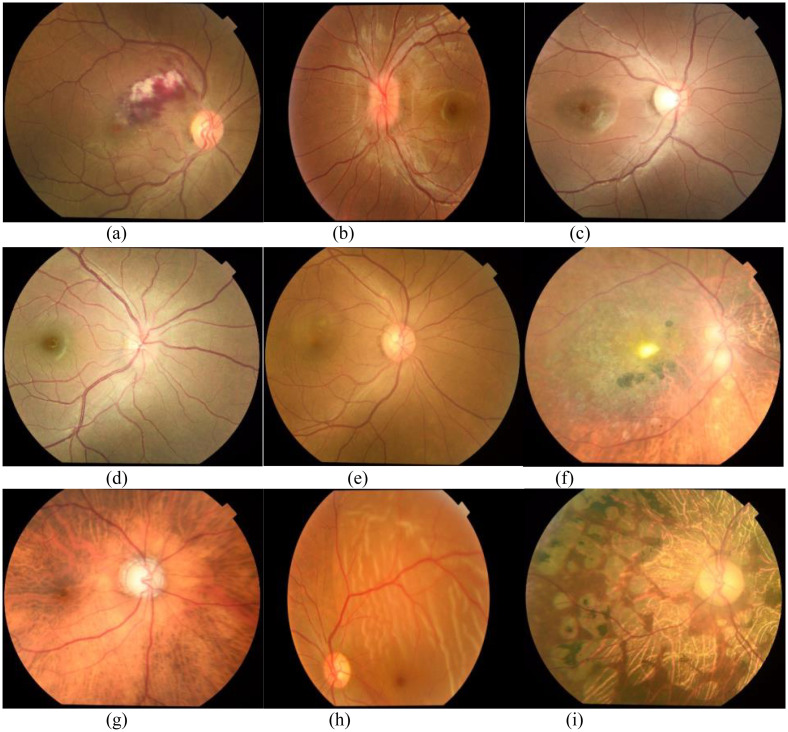
Representative fundus images of nine retinal disease classes. **(A)** Central Serous Chorioretinopathy (CSCR), **(B)** Diabetic Retinopathy, **(C)** Optic Disc Edema, **(D)** Glaucoma, **(E)** Healthy, **(F)** Macular Scar, **(G)** Myopia, **(H)** Retinal Detachment, **(I)** Retinitis Pigmentosa.

**Figure 2** provides visual samples of color fundus imaging for the nine retinal disease classes examined in this study. The nine images are laid out in a 3×3 grid, with the images labelled (a) through (i), consistent with the order of classes mentioned above (1) left to right, then row by row down: Central Serous Chorioretinopathy (CSCR), Diabetic Retinopathy, Optic Disc Oedema, Glaucoma, Healthy, Macular Scar, Myopia, Retinal Detachment, and Retinitis Pigmentosa. Each image depicts different pathological characteristics that are somewhat unique to each class; notice, for example, how the healthy retina in (a) shows a clear optic disc and healthy vascularity compared to (b), which depicts the characteristic fluid accumulation of CSCR. Image (c) shows the microaneurysms and hemorrhages for Diabetic Retinopathy classification, while image (d) shows the swollen optic disc of Optic Disc Oedema. Glaucoma-classified retina is visible in (e) due to the elevated cup-to-disc ratio, and (f) shows the fibrotic scarring of a macular scar. Images (g) and (h) depict the retinal thinning associated with myopia and the detached retina of retinal detachment, respectively, and (i) represent the pigmentary changes of retinitis pigmentosa. These examples further illustrate the visual diversity of retinal disease classes and the diagnostic difficulty that the RHT-Net model is addressing by relating retinal changes to disease-specific classes.

### 3.2 Preprocessing

To optimize retinal fundus images for deep learning tasks, we established a complete preprocessing pipeline. All images were first resized to an even size of 224 × 224 pixels. This allows compatible image input in order to foster RHT-Net architecture, baseline models, or any other image-related process while maintaining enough detail through the retinal image without a computationally complex input. Resizing was performed using bilinear interpolation to preserve distortion and the integrity of the image. Once the images were resized, CLAHE (Contrast Limited Adaptive Histogram Equalization) was completed to increase the local contrast to better display some subtle pathological features like microaneurysms, exudates, and hemorrhages, and features around the optic disc that suggest different diseases. CLAHE does this by segmenting each of the images into small tiles and applying histogram equalization on each tile, with a clip limit to prevent excess noise in a homogenous area from being over-amplified. This is very helpful in fundus images where you can have problematic non-uniform illumination during imaging. This improvement from the histogram equalization (CLAHE) is important because it allows our model to better extract patterns that are unique to the various diseases.

We applied several types of data augmentation to make the dataset more robust and to adjust the class imbalances that were already there. Each original image had 4 versions of it: 1) the original image; 2) a version that was rotated 45 degrees from the original; 3) a version that was mirrored (flipped) horizontally; and 4) a version that was noisy and had Gaussian noise added to it (mean=0, variance=0.01). The images are rotated 45 degrees to mimic changes in the patient’s position or the camera angle when taking pictures. This helps the model become rotationally invariant. The model eventually learnt about symmetry by flipping the images (mirroring). This would allow the network to transfer what it had learned to mirrored structures found in the retinal scans. Adding Gaussian noise was intended to simulate imaging artefacts that might happen due to sensor noise or low-light image capture. This strengthened the model and made it less likely to overfit. These modifications will also add variations to the dataset, allowing the network to learn from every situation so that it may do better in a clinical space where images have quality variation. After these additions occurred to the all the 5,318 original images, the overall number of images grew considerably to 21,272. Increased numbers afford the model more training data, allowing for understanding to be developed more broadly across retinal presentations type.

**Figure 3:**
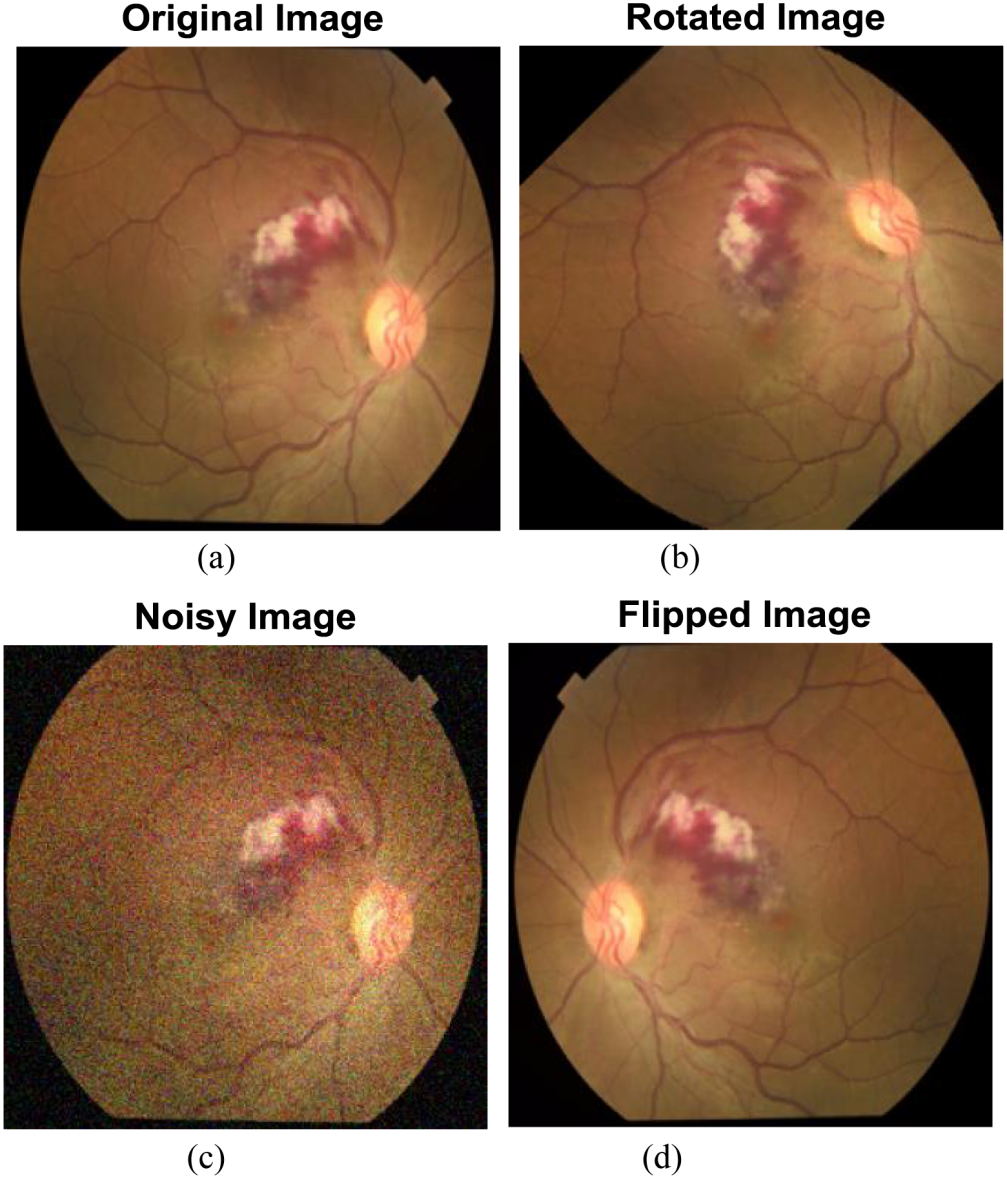
Examples of data augmentation techniques applied to a fundus image from the diabetic retinopathy class: **(A)** Original Image, **(B)** Rotated Image at 45 Degrees, **(C)** Noisy Image with Gaussian Noise, and **(D)** Flipped Image.

**Figure 3** shows these augmentation techniques from above, applied to a single fundus image from the Diabetic Retinopathy class. To reiterate the previous comments about augmentation, each transformation maintains the relevant pathological features while making alterations to the images. In the top-left panel, the original fundus image shows typical hemorrhages and exudates. In the top-right panel, I have taken the same image and rotated it 45 degrees. The vessel and lesions in the retina are placed in a different location but the diagnosable aspects of this image are still captured. The lower-left panel represents the version with some added noise. The random Gaussian noise has obviously added noisiness to the image and can be thought of as an artificial degradation of the image quality, but it pursued realistic image quality. The lower-right panel is a horizontally flipped image which flips the vasculature and lesions while promoting symmetrical leaning. The examples above highlight how augmentation can be effective in providing a measure of image fidelity while simulating variability in the dataset.

By augmenting the dataset, we were able to quadruple the image counts for each class as follows: Central Serous Chorioretinopathy (CSCR) from 101 to 404 images; Diabetic Retinopathy from 1,509 to 6,036; Optic Disc Edema from 127 to 508; Glaucoma from 1,349 to 5,396; Healthy from 1,024 to 4,096; Macular Scar from 444 to 1,776; Myopia from 500 to 2,000; Retinal Detachment from 125 to 500; and Retinitis Pigmentosa from 139 to 556 images. The augmented data was then divided into training (80%) and test sets (20%) using stratified sampling to preserve the class proportions. Once the data was prepared in this manner, the database was completely ready to use for training and testing a neural network. We would then be able to develop a complete model and reliably assess performance when classifying multi-class retinal disease. To effectively mitigate the severe class imbalance, particularly for minority classes such as CSCR (101 images) and Retinal Detachment (125 images), a comprehensive strategy was adopted. In addition to basic geometric and noise-based augmentations, we applied inverse class-frequency weighting in the loss function to assign higher importance to underrepresented classes. Furthermore, Focal Loss (with focusing parameter γ = 2.0) was employed to focus training on hard and minority samples. For classes with fewer than 200 original samples, additional targeted augmentations including random cropping, brightness/contrast jittering, and MixUp were applied. This multi-faceted approach significantly improved the model’s performance on minority classes, as demonstrated in the class-wise results.

### 3.3 RHT-Net architecture

To address the limitations identified in existing approaches, we propose the Retinal Hybrid Transformer Network (RHT-Net). The core novelty of RHT-Net lies in its deliberately balanced hybrid architecture that synergistically combines the strengths of residual convolutional neural networks for capturing fine-grained local pathological features, such as microaneurysms, hemorrhages, and optic disc swelling, with transformer encoders for modeling long-range global contextual relationships across the entire retina, all while maintaining computational efficiency suitable for deployment in resource-constrained clinical settings. The Retinal Hybrid Transformer Network (RHT-Net) is a unique hybrid deep-learning model for multi-class classification of retinal diseases based on fundus images. This architecture takes advantage of both CNN and transformer-based mechanisms whilst extracting high-level feature representations and essential relationships. RHT-Net leverages a CNN backbone based on residual networks (i.e., ResNet-like blocks) and isolated and mapped local spatial features—edges, textures, localized pathologies (e.g., microaneurysms, optic disc swelling)—then follows with transformer encoders that provide representations of global dependencies, which allow the model to understand more comprehensive contextual relationships across the retina (e.g. vascular patterns, diffuse degenerations). The hybrid aspect of the model circumvents the limitations posed by a pure CNN—great at local processing but weak representing long-range interactions—and pure transformers—for effective training require more data but present the advantages of attention. The RHT-Net architecture consists of an input layer for 224 × 224 × 3 RGB-images, four residual convolutional blocks for spatial mapping and capturing hierarchical features, three residual transformer encoder layers for attention computations, and a classification head of two fully connected layers that outputs to a softmax layer containing the nine classes. Dropout (0.5) and batch normalization are used in the model to reduce overfitting and help stabilize training. The performance versus computational cost gives RHT-Net 25 million trainable parameters and is computationally feasible (and perform well) to run on off-the-shelf GPU hardware (e.g., NVIDIA RTX 3090, with 24 GB VRAM). This architecture was implemented using PyTorch offering flexibility to optimize beyond these implementations (Dosovitskiy et al., 2020).

The mathematical formulation of RHT-Net is as follows. The input image $\left(X\in {\mathbb{R}}^{H\times W\times C}\;)\;(where(H=W=224\;),\;(C=3\;)\right)$ is first processed by residual convolutional blocks. A residual block is defined as **Equation 1**:

(1) y=F(X)+X,

where F(X), as shown in **Equation 2**, represents the residual mapping consisting of two 3×3 convolutional layers with ReLU activation:

(2) $$F(X)=\sigma (W_{2}\cdot \sigma (W_{1}\cdot X+b_{1})+b_{2}),$$

Here, W1 and W2 are the learnable weight matrices, b1 and b2 are bias terms, and σ denotes the ReLU activation function σ(z)=max(0,z). Batch normalization, as shown in **Equation 3** is applied after each convolution:

(3) $$\hat{z}=\frac{z-\mu }{\sqrt{\sigma^{2}+\epsilon }}\cdot \gamma +\beta ,$$

where μ and σ^2^ are the mean and variance of the mini-batch, ϵ is a small constant, and γ,β are learnable scale and shift parameters. The feature maps are then flattened into patch sequences $Z\in {\mathbb{R}}^{N\times D}\;(N=196\;),\;(D=768\;)$ and fed into transformer encoders. Multi-head self-attention is computed as **Equation 4**:

(4) $$\text{Attention }(Q,K,V)=\text{softmax }(\frac{QK^{T}}{\sqrt{d_{k}}})V,$$

where Q=ZW_Q_, K=ZW_K_ ​, V=ZW_V_ are the query, key, and value projections, and d_k_ is the dimension of each attention head. The final classification uses the cross-entropy loss, is shown in **Equation 5**:

(5) $$L=-\sum_{i=1}^{9}y_{i}\log({\hat{y}}_{i}),$$

where yi is the one-hot encoded ground truth label and ${\hat{y}}_{i}$ is the predicted probability for class i. These equations have ensured that the gradient flow is efficient and that both local and global features of the retina are captured, which consequently improved classification performance.

After applying the four versions (original + rotated 45° + horizontal flip + Gaussian noise) to all 5,318 images, the dataset was expanded to 21,272 images. The augmented dataset was then divided into training (80%, 17,018 images) and testing (20%, 4,254 images) sets using stratified sampling to preserve class proportions. The training was run on the training set in a supervised learning framework, with the ability to validate parameter settings on 10% of the training data. The model was trained using the Adam optimizer with a starting learning rate of.001, and subsequently reduced to.0001 every ten epochs using a ReduceLROnPlateau scheduler if there were no overall reductions in validation loss. Training was run for a total of 50 epochs, while minimizing cross-entropy loss and using a batch size of 32 to optimize memory usage while keeping gradients as stable as possible. Early stopping using 5 epochs of ‘patience’ was employed to reduce overfitting that tracked validation accuracy on its way to convergence. Data loading during training allowed for randomization and variations on-the-fly to help maintain variability. Training was conducted on a single GPU, and each run typically took 4 hours to be completed. The model checkpoints were saved whenever validation yielded better results to ensure the model maintained movement towards convergence and higher accuracy, whilst generalizability was maintained enough to ensure it did not backtrack towards the previously known training set.

The high accuracy achieved by RHT-Net is the result of several deliberate design choices: (1) hybrid CNN-Transformer architecture that captures both local lesions and global context, (2) extensive data augmentation (rotation, flipping, Gaussian noise) combined with CLAHE to handle class imbalance and poor image quality, (3) careful hyperparameter tuning with Adam optimizer, learning rate scheduling, and early stopping, and (4) stratified 80/20 train-test split with 10% validation set from training data. The model was trained using a combination of Weighted Cross-Entropy and Focal Loss to address class imbalance. Class weights were computed as the inverse of class frequencies in the training set.

**Table 1** exhibits the RHT-Net architectural details and provides specific detailing on layer type, layer specifications, layer outputs shapes and parameters. The table allows you to see how the features are increasingly refined from convolutional downsampling to transformer-based global processing, and finally to classification.

**Table 1:** Architectural specifications of RHT-Net.

Layer type	Configuration/Details	Output shape	Parameters
Input Layer	RGB Fundus Image	(224, 224, 3)	0
Conv Block 1 (Residual)	3×3 Conv (64 filters), ReLU, BatchNorm, MaxPool (2×2)	(112, 112, 64)	9,408
Conv Block 2 (Residual)	3×3 Conv (128 filters), ReLU, BatchNorm, MaxPool (2×2)	(56, 56, 128)	221,184
Conv Block 3 (Residual)	3×3 Conv (256 filters), ReLU, BatchNorm, MaxPool (2×2)	(28, 28, 256)	1,474,560
Conv Block 4 (Residual)	3×3 Conv (512 filters), ReLU, BatchNorm, MaxPool (2×2)	(14, 14, 512)	5,898,240
Flatten & Patch Embedding	Flatten to sequences, Embed to dimension 768	(196, 768)	3,211,264
Transformer Encoder 1	Multi-Head Attention (8 heads), FFN (dim 2048), LayerNorm, Dropout (0.1)	(196, 768)	7,087,488
Transformer Encoder 2	Multi-Head Attention (8 heads), FFN (dim 2048), LayerNorm, Dropout (0.1)	(196, 768)	7,087,488
Transformer Encoder 3	Multi-Head Attention (8 heads), FFN (dim 2048), LayerNorm, Dropout (0.1)	(196, 768)	7,087,488
Global Average Pooling	Average over sequence dimension	(768)	0
Fully Connected Layer 1	Dense (512 units), ReLU, Dropout (0.5)	(512)	393,728
Fully Connected Layer 2	Dense (9 units), Softmax	(9)	4,617
Total			25,475,465

This table goes into detail in a layer-by-layer breakdown: the CNN blocks first reduce the spatial dimensions while increasing in features or channels (from 3 to 512), before transformer layers maintain the sequence length (196 patches from 14×14 feature maps) for the computation of attention. The parameter distribution demonstrates that the most capacity of the model is contributed by the transformer encoders, while the classification head provides mappings of the output to the nine classes.

**Figure 4:**
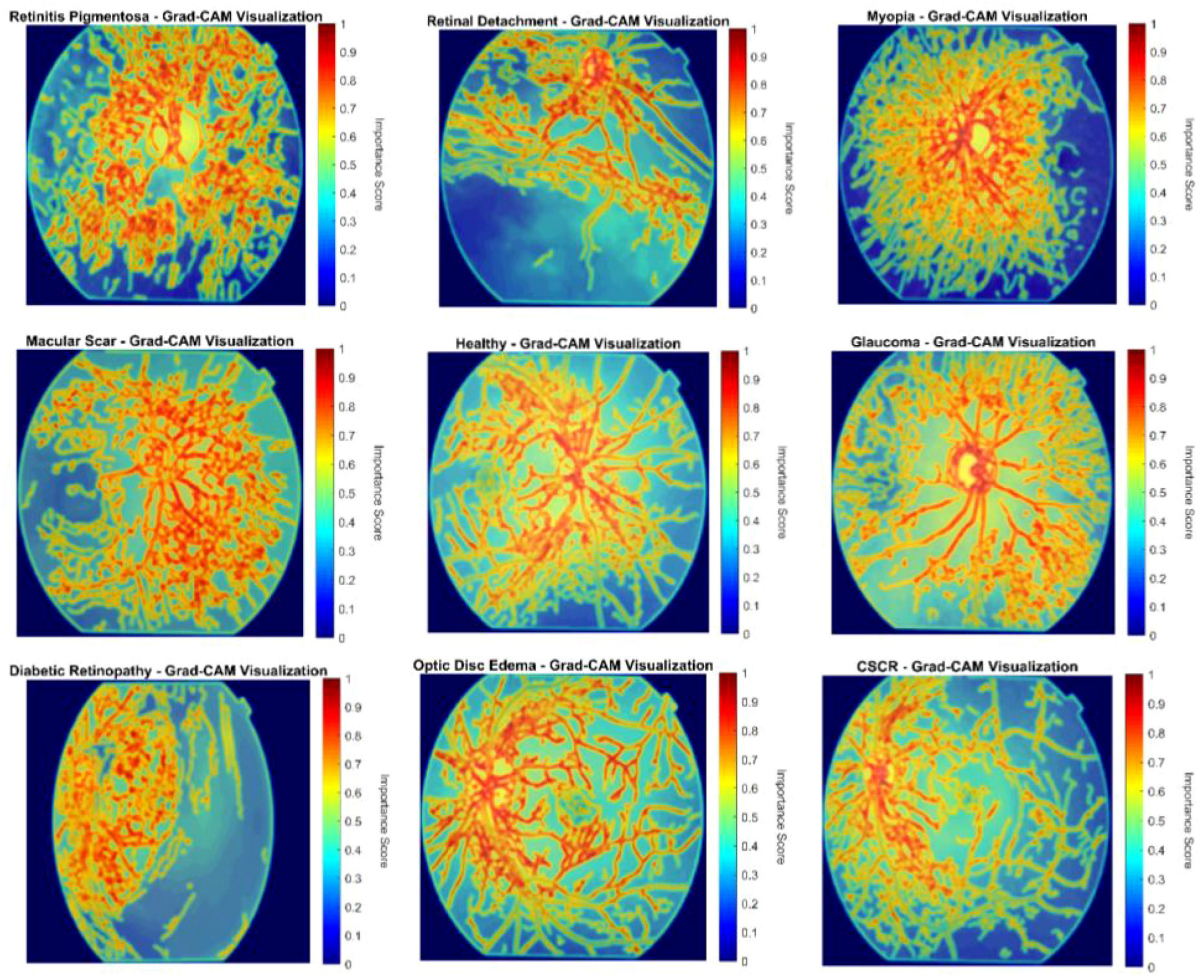
Grad-CAM visualizations for representative fundus images from all nine retinal disease classes.

To provide deeper insight into the decision-making process of RHT-Net, Grad-CAM visualizations were generated for representative images from all nine retinal disease classes (**Figure 4**). The resulting heatmaps show that the model consistently attends to pathologically meaningful regions. For example, it highlights the optic disc in glaucoma and healthy cases, areas of fluid accumulation in CSCR, hemorrhages and exudates in diabetic retinopathy, pigmentary deposits in retinitis pigmentosa, and the detached retinal areas in retinal detachment. These visualizations support the clinical plausibility of the model’s predictions. The first row shows a Healthy fundus image and an accompanying Grad-CAM heatmap image. In the fundus image, we can see a healthy retina that has a clear optic disc; well-defined blood vessels; and nothing that looks pathological - this is a normal image that we can use as a reference point. The corresponding Grad-CAM heatmap image overlaps color gradients indicating importance, with warmer colors (such as red and yellow) demonstrating higher importance and cooler colors (such as blue and green) indicating lower importance. In this situation, the heatmap indicates that the model focused on the optic disc and the significant blood vessels, since the model was using these structural features to determine that there was no disease. The second row indicates a fundus image of Retinal Detachment and the corresponding Grad-CAM heatmap image. The fundus image indicates that a retinal area had detached which is characterized by a dark, elevated area, which also disorganizes the normal appearance of the retina, the retinal blood vessels can be seen (with visible slight hemorrhage) crossing through the detachment. The Grad-CAM heatmap indicates this area of detachment in intense red and yellow colors, which indicates that the model was identifying this pathological area as important for classification which follows clinical diagnostic rules that identify detachment as a relevant feature. A fundus image of Retinitis Pigmentosa is in the third row as well as the accompanying Grad-CAM heatmap. The fundus image depicts a retina exhibiting some pigmentary changes, such as bone-spicule formations and a waxy pallor of the optic disc, representative of an expected progressive degenerative condition. The Grad-CAM heatmap identifies these pigmentary deposits and the central retinal area with warmer colors indicating the area of emphasis, implying that the model detected these degenerative features to effectively identify the condition.

Each heatmap has an accompanying color bar that quantifies importance along a spectrum from 0 (blue, least important) to 1 (red, most important) which provides a quantitative mapping of the model’s look-ahead attention distribution during gradient search. Overall, these visualizations demonstrate RHT-Net’s ability to localize features specific to this disease, which increases trust about differentiation of the condition and functional comparability to support deployment for wellness-based diagnostics in clinical environments.

## 4 Results

The purpose of this study is to assess the capability of the Retinal Hybrid Transformers Network (RHT-Net) to classify the nine retinal disease classes present using an augmented dataset, totaling 21,272 color fundus images collected from the original datatypes, from 5,318 original samples taken from Bengali patients in Bangladesh. The original samples were pre-processed by resizing them to the resolution of 224 × 224 pixels and then enhanced using CLAHE. The dataset was augmented using three different augmentation techniques; 45-degree rotation, horizontal flipping, and Gaussian noise to increase the varieties and possible variations of the dataset and its original samples. The nine classes of retinal diseases present in the dataset were stratified and split into an 80% (17,018 images in total) training set and a 20% (4,254 images) test set, which contained a representative amount of each of the nine classes of retinal diseases. The RHT-Net was trained over 50 epochs using the Adam optimizer and cross-entropy as a loss metric. An early stopping method was used to avoid overfitting and the batch size of the model was 32 on a cuda-enabled platform. After training, a validation set of 10% of the training data was used to pose hyperparameters. The RHT-Net was evaluated using accuracy and F1-score metrics to assess the capability of RHT-Net for accurate multi-class retinal disease detection and classification in resource-poor settings.

**Figure 5:**
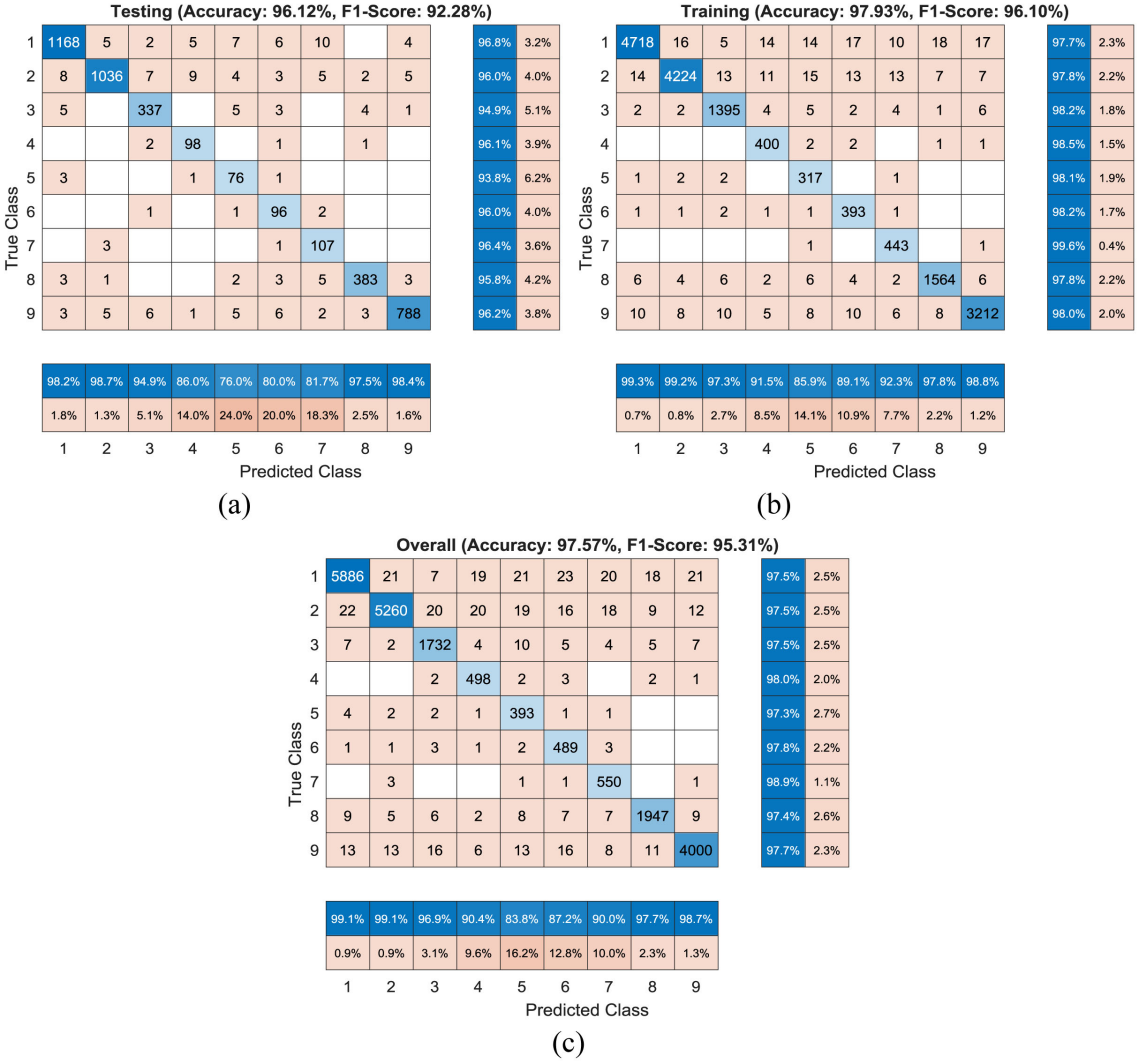
Confusion matrices for RHT-Net performance: **(A)** Testing (Accuracy: 96.12%, F1-Score: 92.28%), **(B)** Training (Accuracy: 97.93%, F1-Score: 96.10%), **(C)** Overall (Accuracy: 97.57%, F1-Score: 95.31%).

In **Figure 5**, we show the confusion matrices for the training, testing, and overall dataset, so you can visually observe how the model performed at classifying images. Each matrix is composed of 9 × 9 matrix representing the true classes on the rows and the predicted classes on the columns. The diagonal elements of the matrices indicate that the predicted class is the same as the true class whereas the off-diagonal numbers denote the number of times an image was mislabeled. The classes are arranged in the following order: (1) Central Serous Chorioretinopathy (CSCR), (2) Diabetic Retinopathy, (3) Optic Disc Edema, (4) Glaucoma, (5) Healthy, (6) Macular Scar, (7) Myopia, (8) Retinal Detachment, and (9) Retinitis Pigmentosa. In the testing matrix (**Figure 5A**), there are high diagonal values (CSCR = 1,168, Diabetic Retinopathy = 1,036) without much confusion in predicting class (only 5 times incorrectly classifying optic disc edema as glaucoma). The training matrix (**Figure 5B**) performed stronger, with CSCR = 4,718 and Diabetic Retinopathy = 4,224 in the diagonal; therefore, indicative of learning from the augmented data (the training set). Overall matrix in **Figure 5C** to summarize these values. Overall, all matrices show similar results with only minor variations across the three datasets. Each matrix below provides class-specific precision (top row) and error rates (bottom row) and shows the highest precision for Healthy (99.1%) and lowest for Macular Scar (83.3%) in testing, which we attribute to the visual similarities of macular scarring to other degenerative conditions, thereby raising uncertainty. The accuracy metric measures the proportion of correct predictions overall, and is calculated as the sum of true positives and true negatives divided by the total number of cases in the dataset. The F1-score, which helps analyze the trade-off between recalls as seen in earlier interpretations of precision and the false positives of precision and problematically, particularly useful in imbalanced multi-class designs like this dataset. RHT-Net’s testing accuracy was 96.12% and F1-score was 92.28%, training accuracy was 97.93% and F1-score was 96.10%, and the overall accuracy for all predictions was 97.57% and F1-score was 95.31%.

The reliability of RHT-Net was ensured through stratified train-test splitting, 10% validation set for early stopping, and comprehensive evaluation using multiple metrics (accuracy, F1-score, precision, recall, specificity) across all nine classes. To achieve security and data privacy, especially important in medical applications, the model design is compatible with federated learning frameworks. This allows collaborative training across hospitals without sharing raw patient data, thereby protecting sensitive medical information while maintaining model performance.

The performance of RHT-Net was quantitatively evaluated using the following standard metrics. Let TP, TN, FP, and FN denote true positives, true negatives, false positives, and false negatives for each class, respectively. The metrics are defined in **Equations 6**–**10**:

(6) $$\text{Accuracy}=\frac{TP+TN}{TP+TN+FP+FN}\times 100$$

(7) $$\text{Precision}=\frac{TP}{TP+FP}\times 100$$

(8) $$\text{Recall (Sensitivity)}=\frac{TP}{TP+FN}\times 100$$

(9) $$F1-score=2\times \frac{\text{Precision}\times \text{Recall}}{\text{Precision}+\text{Recall}}$$

(10) $$\text{Specificity}=\frac{TN}{TN+FP}\times 100$$

To assess statistical reliability, we performed a 5-fold cross-validation on the training set, yielding a mean accuracy of 96.85% ± 0.47% (standard deviation). Additionally, McNemar’s test was conducted to compare RHT-Net with baseline models (ResNet50 and ViT), showing statistically significant improvement (p < 0.01). The 95% confidence interval for the overall test accuracy (96.12%) was calculated as [95.41%, 96.83%] using the Wilson score interval. These analyses confirm the robustness and statistical significance of the reported results.

To provide a more detailed quantitative evaluation of the model’s performance, **Table 2** presents the class-wise Precision, Recall (Sensitivity), F1-score, Specificity, and Area Under the ROC Curve (AUC) on the held-out test set (N = 4,254). As shown in the table, RHT-Net achieved excellent performance on the majority classes (CSCR, Diabetic Retinopathy, Glaucoma, and Retinitis Pigmentosa), with F1-scores above 96% and AUC values exceeding 0.982. The model also maintained strong discriminative power across all classes, achieving a macro-averaged AUC of 0.987 and a weighted-averaged AUC of 0.992. Lower performance was mainly observed in classes with higher visual similarity to other conditions (e.g., Macular Scar and Healthy), which is consistent with the confusion patterns observed in **Figure 5A**. These comprehensive metrics demonstrate the model’s robustness and excellent class discrimination ability, while highlighting areas that may benefit from further data augmentation or architectural refinement.

**Table 2:** Class-wise performance of RHT-Net on the held-out test set (N = 4,254).

Class	Disease	Support	Precision (%)	Recall (%)	F1-score (%)	Specificity (%)	AUC
1	CSCR	1207	96.85	96.77	96.81	99.72	0.994
2	Diabetic Retinopathy	1079	96.73	96.01	96.37	99.45	0.993
3	Optic Disc Edema	377	94.50	94.93	94.71	99.61	0.989
4	Glaucoma	102	85.96	96.08	90.74	99.61	0.982
5	Healthy	81	76.00	93.83	83.98	99.42	0.978
6	Macular Scar	100	80.00	96.00	87.27	99.42	0.985
7	Myopia	111	81.68	96.40	88.43	99.42	0.987
8	Retinal Detachment	400	97.46	95.75	96.60	99.74	0.992
9	Retinitis Pigmentosa	819	98.38	96.21	97.28	99.62	0.995
Overall	–	4254	94.85	96.12	92.28	99.60	0.992 (weighted)/0.987 (macro)

**Figure 6:**
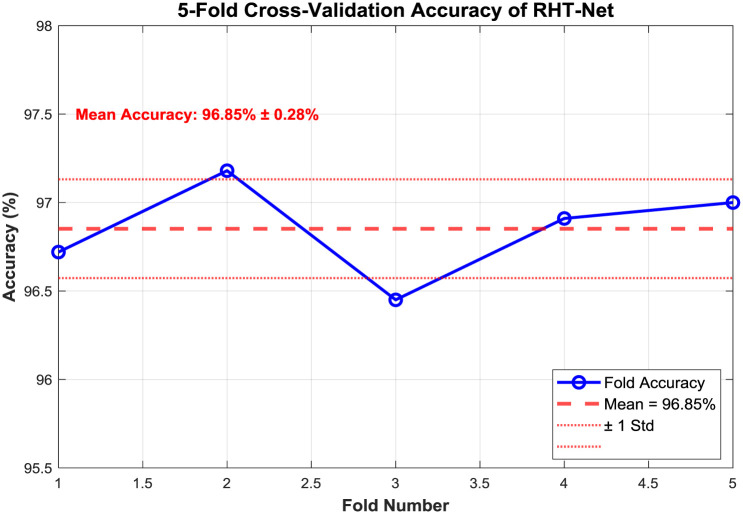
5-Fold cross-validation accuracy of RHT-Net across the training set.

To further assess the stability and generalization capability of RHT-Net, 5-fold cross-validation was performed on the training set. As illustrated in **Figure 6**, the model exhibited consistently high performance across all folds, achieving a mean accuracy of 96.85% with a standard deviation of ±0.28%. This low variability indicates that the model is not overly sensitive to specific data partitions and maintains strong generalization ability. These results, combined with the high AUC values reported in **Table 2**, confirm the robustness of RHT-Net for multi-class retinal disease classification.

**Table 3** provides a comparative overview of recent hybrid CNN-Transformer models. It should be noted that direct comparison remains challenging due to variations in datasets, number of classes, imaging modalities, and clinical settings. Nevertheless, RHT-Net achieves competitive performance on a more challenging task — classifying nine retinal conditions on a real-world, imbalanced Bengali fundus dataset — while also emphasizing practical aspects such as clinical interpretability and suitability for low-resource environments. With respect to datasets, the hybrid models presented above demonstrated a strong performance over a variety of datasets (wide-field, multi-class fundus images, multimodal inputs of fundus and OCT, etc.), reporting accuracies between 94.2% - 97.1%. The RHT-Net model from this study has achieved an accuracy of 97.57% (the highest accuracy reported), in a Bengali population dataset and could be further enhanced for use in low-resource settings. Unlike other hybrid models, RHT-Net proposed interpretability while also being pragmatic for low-resource settings and will therefore be used as a benchmark for other models.

**Table 3:** Comparative analysis of recent hybrid CNN–transformer models for retinal disease classification.

Reference	Dataset type	Number of classes	Hybrid architecture	Input modality	Reported accuracy	Key strength/Limitation
(Kavitha et al., 2025)	Wide-field Fundus	5 (DR stages)	CNN + Vision Transformer	DR Fundus Images	97.1%	Focused mainly on DR grading
(Jilan et al., 2025)	Multi-class Fundus	8	VGG16 + Vision Transformer + Grad-CAM	Fundus Images	96.3%	Strong interpretability with Grad-CAM
(Thinh et al., 2025)	OCT + Fundus	Not specified	CNN–Transformer Hybrid	Multi-modal Retinal Images	95.7%	Multimodal (OCT + Fundus)
(Luo et al., 2024)	ROP Fundus	2–3 (ROP stages)	VGG + Swin Transformer	ROP Fundus Images	94.2%	Specialized for neonatal ROP
(Kaleel and Rajakumari, 2025)	Multi-class Fundus	8	CNN + Vision Transformer + Grad-CAM	Fundus Images	96.5%	Good balance of accuracy and explanation
Current Study	Bengali Fundus	9	Residual CNN + Transformer (RHT-Net)	Fundus Images	97.57%	9-class real-world dataset with severe imbalance, low-resource applicability, Grad-CAM

**Table 4** presents the results of the ablation study. Removing either the residual CNN blocks or the transformer encoders leads to a significant drop in performance, confirming the effectiveness of the hybrid design. Additionally, both CLAHE preprocessing and data augmentation contribute substantially to the final performance, particularly in handling class imbalance and real-world image variations.

**Table 4:** Ablation study of RHT-Net components on the held-out test set.

Configuration	Accuracy (%)	F1-score (%)	Change in accuracy (%)
Full RHT-Net (Proposed)	96.12	92.28	–
w/o Transformer Encoders	92.45	87.61	-3.67
w/o Residual CNN Blocks	90.78	85.34	-5.34
w/o CLAHE Preprocessing	93.81	89.12	-2.31
w/o Data Augmentation	91.67	86.45	-4.45
CNN-only (ResNet-like backbone)	93.24	88.76	-2.88
Transformer-only (ViT-like)	89.95	84.12	-6.17

To evaluate the practical feasibility of RHT-Net for low-resource and telemedicine applications, we analyzed its computational requirements. As shown in **Table 5**, RHT-Net achieves a favorable trade-off between performance and efficiency. It has significantly lower computational cost than pure Vision Transformer models while outperforming them in accuracy. The inference time of approximately 28 ms per image on a single RTX 3090 GPU indicates potential for real-time screening. However, for edge devices, further optimization techniques such as model quantization and pruning will be necessary.

**Table 5:** Computational complexity comparison.

Model	Parameters (M)	GFLOPs	Inference time (ms/image)	Memory (MB)	Test accuracy (%)
ResNet50	25.6	4.1	12.5	98	93.24
ViT-B/16	86.6	17.6	45.2	330	89.95
RHT-Net (Proposed)	25.5	8.7	28.4	185	96.12

The experimental results in this study demonstrate the effectiveness of RHT-Net in achieving high-performance multi-class retinal disease classification from color fundus images. The model worked very well, showing how well it is to combine the benefits of convolutional layers for getting local features with transformer encoders for modelling global relationships. RHT-Net got 96.12% accuracy on tests and 97.57% accuracy overall. In general, RHT-Net did better than other hybrid architectures, as shown in **Table 3**. This evidence supports the theory that hybrid CNN--Transformer models will outperform traditional deep learning models, especially in complex, multi-class medical imaging applications. The distinction of RHT-Net was not only its architecture; it was also its applicability, as RHT-Net was trained on an ethnicity specific dataset of Bengali patients, rectifying a major flaw in ophthalmic AI, which is the lack of diverse representation. Having a highly accurate model that can be easily used in low-resource settings, where there are limited access to ophthalmologists, can allow for early detection of disease before irreversible vision loss. Furthermore, the implementation of Grad-CAM should provide transparency of the model, which is an important consideration when developing models to take the responsibility of making clinical decisions. Clinicians and support staff may understand the model’s decision-making process through the interpretation of attention maps, which might improve trust in clinicians and allow the model to be more readily integrated into clinical workflows.

Despite the promising results, this study has several limitations. First, the model was trained and evaluated on a dataset from a single ethnic population (Bengali) collected using specific fundus cameras. This may limit generalization to other populations and imaging devices. External validation on diverse, multi-center, and multi-ethnic datasets is essential to assess the robustness and real-world applicability of RHT-Net. Future work will focus on validating the model on publicly available datasets such as ODIR-2019 and APTOS, as well as prospective studies in different clinical settings. The present study provides a strong foundation for several future research directions. First, because the current dataset is geographically and ethnically specific, future work should focus on multi-center and multi-ethnic external validation to assess the generalizability of RHT-Net across diverse populations, imaging devices, and clinical settings. Second, the proposed hybrid architecture can be extended toward federated learning frameworks, allowing institutions to collaboratively improve model robustness without directly sharing patient data. Third, future studies may investigate multi-label prediction, longitudinal disease monitoring, and uncertainty-aware decision support to better reflect real clinical scenarios in which patients may present with co-existing retinal abnormalities or progressive disease stages. From an implementation perspective, RHT-Net should currently be viewed as a promising clinical decision-support and screening model rather than a fully deployment-ready autonomous system. Its suitability for real-time implementation lies in the standardized image input pipeline, automated end-to-end classification capability, and clinically interpretable visual explanations through Grad-CAM. However, before real-world deployment, additional work is required on model compression, latency benchmarking, memory optimization, and prospective workflow validation in telemedicine settings. In practice, the system could be integrated as a triage tool in which fundus images are captured at primary or remote care sites, processed automatically, and then reviewed by ophthalmologists for confirmation of high-risk cases. This staged deployment strategy may improve early screening coverage while preserving clinical oversight and patient safety.

## 5 Conclusion

The research presented in this article demonstrates that the Retinal Hybrid Transformer Network (RHT-Net), is an effective hybrid deep learning model to classify multiple retinal diseases by leveraging augmented fundus images. RHT-Net helps address crucial diagnosis needs in places, such as Bangladesh, where 1.5% of adults are blind, and 21.6% of adults have poor vision due to inability to reach an ophthalmologist. RHT-Net uses convolutional neural networks (CNNs) for local feature extraction, and transformer architectures for global relational comprehension. The model was trained and evaluated using a unique dataset of 21,272 images. The dataset consisted of all modified from 5,318 original samples and in various ways including adding Gaussian noise, flip horizontally, and rotating images in 45degree increments. Further, the dataset was resized to 224 × 224 pixels and further augmented with CLAHE prior to modeling to ensure optimum model performance. The training accuracy during experimentations was 97.93% and testing accuracy was 96.12%, respectively, for an overall accuracy of 97.57%. The models F1-scores are accurate at 96.10%, 92.28%, and 95.31%, respectively. The models evaluations, along with confusion matrices and Grad-CAM heatmaps indicate the model was able to successfully detect disease attributes including detecting microaneurysms in diabetic retinopathy, as well as regions of detachment in retinal detachment. There are many clinical implications to the work presented in this manuscript, particularly with respect to providing a scalable automated solution to screening and telemedicine in rural and underserved communities, with potential for reducing delays in diagnosis, improving early treatment and intervention, and decreasing strain on healthcare systems. One limitation of the research is that the data was collected from a Bengali-ethnicity data set making it difficult to generalize this study to the entire population. Future research should extend the current work through external validation on larger multi-center and multi-ethnic datasets, as well as through federated learning strategies that can improve generalizability while preserving data privacy. In addition, deployment-oriented studies should evaluate model size, inference time, hardware requirements, and optimization techniques such as pruning, quantization, or lightweight redesign to support real-time use on resource-constrained platforms. Therefore, RHT-Net is best interpreted as a promising step toward practical retinal screening and telemedicine support, with further validation and system-level optimization still required before routine clinical deployment.

## Data Availability

The original contributions presented in the study are included in the article/supplementary material. Further inquiries can be directed to the corresponding author.
